# Use and influence of Delivery and Birth Plans in the humanizing delivery process^1^

**DOI:** 10.1590/0104-1169.0067.2583

**Published:** 2015-07-03

**Authors:** María Suárez-Cortés, David Armero-Barranco, Manuel Canteras-Jordana, María Emilia Martínez-Roche

**Affiliations:** 2 Doctoral student, Facultad de Enfermería, Universidad de Murcia, Murcia, Spain; 3 PhD, Full Professor, Facultad de Enfermería, Universidad de Murcia, Murcia, Spain; 4 PhD, Full Professor, Facultad de Medicina, Universidad de Murcia, Murcia, Spain

**Keywords:** Humanizing Delivery, Personal Autonomy, Decision Making

## Abstract

**OBJECTIVES:**

get to know, analyze and describe the current situation of the Delivery and Birth Plans in our context, comparing the delivery and birth process between women who presented a Delivery and Birth Plan and those who did not.

**METHOD:**

quantitative and cross-sectional, observational, descriptive and comparative cohort study, carried out over two years. All women who gave birth during the study period were selected, including 9303 women in the study.

**RESULTS:**

132 Delivery and Birth Plans were presented during the first year of study and 108 during the second. Among the variables analyzed, a significant difference was found in "skin to skin contact", "choice of dilation and delivery posture", "use of enema", "intake of foods or fluids", "eutocic deliveries", "late clamping of the umbilical cord" and "perineal shaving".

**CONCLUSIONS:**

the Delivery and Birth Plans positively influence the delivery process and its outcome. Health policies are needed to increase the number of Delivery and Birth Plans in our hospitals.

## Introduction

The delivery is a normal and natural process, a vulnerable period for women's health, in which the environment and the health activities exert great influence. In the mid 20^th^ century, the delivery process was institutionalized, changing from the home-based to the hospital-based deliveries. By considering the delivery as a hospital-based process, certain routine and protocoled practices were included, such as episiotomy, shaving, enemas, induction of birth, without any endorsement of their routine use by means of scientific evidence. The delivery was included in the health care model of disease^1^, considering women as ill persons in need of medical care^2^. In view of this situation, in 1985, as a result of WHO's birth recommendations, a process started to standardize the delivery, in which the states were encouraged to reconsider the technology applied to birth, acknowledging that each woman should choose the type of delivery she wants, thus contributing to return the women's protagonist role^3^. In Spain, in the 1990's, professional and women's groups start to consider that delivery care is excessively interventionist^4^ In 1996, WHO in Geneva^5^ published the guide "Normal birth care: a practical guide", giving rise to different documents in defense of normal birth^4^^,^ 6 ^-^ 7. That is the start of women's empowerment, which is demonstrated most clearly in the Delivery and Birth Plan.

The concept of Delivery and Birth Plan was coined by Sheila Kitzinger in the United States in 1980^8^. The Anglo-Saxon countries echoed this new document and started to use it to require delivery with as little medical intervention as possible.

A Delivery and Birth Plan is a written legal document in which the pregnant woman, after having received information about the pregnancy and the delivery procedure, and in view of her personal values and desires, besides the expectations created about her delivery in the course of the pregnancy, and also attending to all of her private needs, agrees with the Primary Health Care midwife, and later with the Hospital midwife, on which alternatives, according to the best praxis, she prefers during her delivery in normal conditions. The Delivery and Birth Plan is the axis of the clinical relation established between the pregnant woman and the midwife, and serves to guide the health care the latter delivers to the former throughout the process.

The importance of the Delivery and Birth Plans derives from the respect for the Bioethical Principle of Autonomy, thus enhancing women's control over the delivery process and contributing to a positive effect on their satisfaction^9^, serving as an important tool to prepare for birth^10^^-^ 11 and reducing the women's "fears" thanks to the information and communication they offer^10^^-^ 15 ^,^; constituting a reflection process for the women^12^^,^ 16.

The pregnant women have always felt the need to plan and inform their families and health professionals about what is important to them, so as to be able to feel safe and support during the birth process^16^.

In addition, it should not be forgotten that pregnancy and delivery are the preliminary steps of motherhood, representing the start of the acquisition of the maternal role. Ramona T. Mercer, the author of the theory "Becoming a Mother", defends that the acquisition of the maternal role is a process that demands psychological, social and physical action from the woman^17^, in which she should be aware of her role as the mother of a creature who needs her care and who depends on her. The woman's decision will affect her creature since pregnancy, which is why she should make the decisions after long periods of reflection, considering her values, beliefs and expectations. 

The use of the Delivery and Birth Plan was rapidly generalized in some European countries. In 1993, in England, it was used in 78% of the delivery rooms. In Spain, they were implemented recently, in 2007. The Care Strategy for Normal Delivery in the National Health System (Ministry of Health and Consumption) 6 and the Normal Birth Initiative (FAME) 4 mention this document, but it is only in February 2012 that the Ministry of Health, Social Policy and Equality publishes a model of the Delivery and Birth Plan. 

Today, the Delivery and Birth Plan is one of the items to value delivery care in the Spanish Health System and in the Comprehensive Women's Health Care Program (PIAM) 18 in the Region of Murcia. Therefore, it is important to know the current situation of the Delivery and Birth Plans in that context, how many women present a Delivery and Birth Plan and whether the development and outcome of the delivery differ between the women who presented a Delivery and Birth Plan and those who did not.

The objective in this study is to get to know, analyze and describe the current situation of the Delivery and Birth Plans in the Region of Murcia, as well as to compare the development and outcome of the delivery between the women who presented a Delivery and Birth Plan and the women who did not.

## Method

A quantitative, cross-sectional, observational, descriptive and comparative cohort study was undertaken between January 2011 and December 2012. The sample consisted of all women who gave birth during the study period at the Clinical Teaching Hospital Virgen de la Arrixaca in Murcia, Spain. The inclusion criterion was having given birth at the Clinical Teaching Hospital Virgen de la Arrixaca in Murcia between January 2011 and December 2012, and not attending to any of the exclusion criteria.

The following exclusion criteria were considered: 

Women with a programmed c-section.

Women who gave birth after less than 37 weeks of pregnancy.

Women whose fetus died before birth.

Multiple births (two or more fetuses).

Incorrect completion of "Delivery room record".

In total, 12,579 births were registered between January 2011 and December 2012, 73.96% (9,303 births) of which were included in the study.

To collect the data, the "Delivery room record" registered in the software Selene was used (Computer program used in Hospitals of the Spanish Health System). The following research variables were adopted: 

Birth year: 2011 or 2012.

Monitoring during delivery: Monitoring during delivery by a person selected by the woman, except for the health professionals. 

Monitoring during dilation: Monitoring throughout or during part of the dilation period by a person selected by the woman, except for the health professionals. 

Skin to skin contact: Takes place immediately after birth and refers to the placement of the infant in the prone position in direct contact with the mother. Favors the regulation of the infant's vital functions.

Choice of dilation and delivery position: The woman elects the posture during the dilation and expulsion periods. 

Enema: Application of an evacuation enema during or before the active period of delivery. Its routine use is discouraged. 

Episiotomy: Procedure in which the health professionals make a cut in the perineum when the head is expulsed. Its systematic use is discouraged. Instead, only the selective use is advised. 

Outcome of the delivery: This variable comprises deliveries using equipment, eutocic delivery and intrapartum c-section.

Intake of foods or fluids: Refers to whether the woman has consumed foods or fluids during the active period of birth.

Start of breastfeeding inside delivery room: It is considered that the infant starts breastfeeding in the immediate post-partum period, i.e. during the first two hours after birth. 

Monitoring: Fetal monitoring or RCTG is the control of the fetal cardiac frequency. 

Oxytocin: Use of synthetic oxytocin during the dilation, expulsion or childbirth period. 

Induced births: Procedure aimed at triggering uterine contractions in pregnant women without an effective labor process. 

Late clamping of umbilical cord: It is considered that the clamping was late when this happens after the pulse in the cord stops or one minute after birth.

Delivery and Birth Plan: The women presented a written Delivery and Birth Plan before giving birth or during the active period of labor. 

Perineal shaving: Shaving of the perineum in hospital. 

Type of anesthesia: This variable comprises epidural, general, local and no anesthesia.

For the statistical analysis of the data, the software SPSS.19 was used. First, descriptive analysis of the sample was performed using frequency tables and percentages of all data collected. Next, to determine what variables are related with the use of the Delivery and Birth Plan, contingency tables were developed using Pearson's χ² test, with a 95% confidence interval and, finally, logistic regression was applied to determine what variables are the most influential.

For the research, the necessary permissions were requested from the Institutional Review Board at the Clinical Teaching Hospital Virgen de la Arrixaca and from the hospital manager.

## Results

In total, 9,303 deliveries were analyzed in 2011-2012, 2.6% (240) of which presented a Delivery and Birth Plan.

In 2011, 2.8% (132) of the 4,618 registered births came with Delivery and Birth Plans. In 2012, the number of Delivery and Birth Plans dropped to 2.3% (108) of the 4,685 births (Table 1).

**Table 1.** Delivery and Birth Plan by Year. Clinical Teaching Hospital Virgen de la Arrixaca, Murcia, Spain, 2011-2012

Delivery and Birth Plan	Year		Total
	2011	2012	
Yes	132	108	240
No	4486	4577	9063
Total	4618	4685	9303

The distribution of the sample according to the weeks of pregnancy is shown in Table 2. As observed, the largest percentage of the sample is concentrated in week 40, with 32.4% (34.1% and 28.78%). 

**Table 2.** Distribution of the sample according to the weeks of pregnancy. Clinical Teaching Hospital Virgen de la Arrixaca, Murcia, Spain, 2011-2012

Weeks of pregnancy	Delivery Plan		Total
	Yes	No	
37	15	593	608
38	39	1394	1433
39	60	2609	2669
40	82	2936	3018
41	41	1483	1524
42	3	48	51
Total	240	9063	9303

**Table 3.** Research variables according to presentation of Delivery and Birth Plan. Clinical Teaching Hospital Virgen de la Arrixaca, Murcia, Spain, 2011-2012

Indicator		Standard Ministry of Health and Consumption	% total births	% total births with delivery and birth plan	P
Delivery plan			2.6	100	
Delivery monitoring			73.9	76.3	0.403
Dilation monitoring			87.7	83.3	0.036
Skin to skin contact		≥80	27.4	60.4	0.001*
Choice of position			48.1	62.5	0.001*
Enema			6.8	10.4	0.027*
Episiotomy			51.3	46.2	0.112
Outcome of delivery					0.018*
	Eutocic		73.8	81.6	
	Instruments	5	23.9	16.2	
	Intrapartum c-sections		2.3	2	
Food and fluid intake			33	42	0.002*
Breastfeeding in delivery room			90	91.1	0.412
Continuous monitoring			99	95	0.001
Oxytocin			86.5	83.7	0.199
Induced births		10	28.7	20.8	0,1
Late clamping of cord			63.1	78.3	0.001*
Perineal shaving			12	16.6	0.023*
Type of anesthesia					0.295
	Epidural	30-80	78.1	73.7	
	General		0	0	
	Local		12.7	16.6	
	No anesthesia		9.2	9.5	

* Statistical significance = p=0.005, expected frequency =5 and residual adjustment =2

In the comparison of the delivery process and its outcome among the women with a Delivery and Birth Plan, the analysis of the contingency table revealed significant differences for seven variables (p≤0.005, expected frequency ≥5 and residual adjustment ≥2). Regarding "skin to skin contact", the total percentage was 27.4%, against 60.41% for the deliveries with a Delivery and Birth Plan. Concerning the "choice of the dilation and birth position", 48.1% against 62.5%. For the "use of enema", 6.8% against 10.4%; for the "intake of foods or fluids", 33% against 42%; for "eutocic deliveries", 73.8% versus 81.66%; for "late clamping of the cord" 63.1% against 78.3%; and for "perineal shaving" 12% against 16.6%, respectively (Table 2). 

After a logistic regression analysis, it was determined that the three most influential variables were "skin to skin contact" (OR=4.26), "election of dilation and delivery period" (OR=1.8) and "perineal shaving" (OR=1.6).

## Discussion

In February 2012, the Spanish Ministry of Health, Social Policy and Equality published a Delivery and Birth Plan model. Nevertheless, the total number of Delivery and Birth Plans dropped by 0.5% between 2011 and 2012 at the Clinical Teaching Hospital Virgen de la Arrixaca (Murcia, Spain).

The EAPN (Normal Birth Care Strategy) 6 in its recommendations and the Ministry of Health in the indicators used in the Birth Quality Assessment in 2011 establish a percentage of 80 % or more of "skin to skin contact" after delivery. In the present data, a significant increase is evidenced by 33.01% (27.4 vs. 60.4; p<0.001) is evidenced among the women who presented a Delivery and Birth Plan when compared to women who did not present this document. Although the percentage of 80% was not surpassed, this increase is important, as skin to skin contact contributes to a better adaptation to the physiological changes in the mother and infant^19^. Similarly, in accordance with the Ministry of Health, Social Policy and Equality, it enhances the "benefits in the psychomotor and emotional maturing of the child, which contributes positively to maintain hormonal and communication dynamics between the mother and infant that is positive for both, facilitating the clutch and the start of exclusive breastfeeding", influencing the satisfaction dynamics. Different organisms recommend this practice: Unicef includes it in step number 4 of the iHan^20^and NICE includes it as a recommended practice in one of its guidelines^21^.

Concerning the "choice of the dilation and delivery posture", the EAPN^6^ and the Ministry of Health defend that, during the expulsion period of the birth process, the woman should choose the position she finds most comfortable, which influences the safety and satisfaction dimensions; in addition, this practice has been endorsed in a Cochrane review^22^. In the present study, satisfactory and significant results were found, as the choice of the position increased by 14.4% in the women who presented a Delivery and Birth Plan (48.1 vs. 62.5; p<0.001). 

"Fluid intake" during delivery is another practice the EAPN^6^ and the Ministry of Health endorsed and recommended, with a significant increase by 9% (33 vs. 42; p=0.002) among the women who presented a Delivery and Birth Plan.

As regards the "late clamping of the umbilical cord", the percentage increased by 15.2% (63.1 vs. 78.3; p<0.001), but the number of deliveries during which this practice is adopted remains insufficient, as WHO recommends its use in all births, considering that, among other benefits, it increases the infant's iron reserves at the age of six months by more than 50%^23^.

One important piece of information obtained in this study is that the rate of "eutocic births" increases from 73.8% to 81.66% (p=0.018) in the group of women who presented their Delivery and Birth Plan. This fact influences the safety and effectiveness dimensions^6^. Concerning the "intrapartum c-sections", no significant drop has been observed, similar to the data obtained in other studies in the same context^24^, although studies developed in other countries have found significant differences for this variable^25^.

As regards unexpected data obtained in our study, the women's demand for "enemas" and "perineal shaving" surprisingly increases by 3.6% (6.8 vs. 10.4; p=0.027) and 4.6% (12 vs. 16.6; p=0.023), respectively, among the women who presented a Delivery and Birth Plan. These two practices are no longer used routinely, but do not involve any harm for the woman or fetus. They only involve the woman's comfort and shame. These data may be due to the fact that the women who presented a Delivery and Birth Plan know their ability to choose and prefer to use these practices for the sake of comfort and to avoid embarrassing situations, such as bowel movements while giving birth.

The study limitations include the lack of completion of forms, which were eliminated to minimize possible bias, as well as the absence of some interesting parameters from the "Delivery room register", which would have broadened the perspective in this study, such as the participation in birth preparation classes, length of dilation and parameters of fetal wellbeing.

## Conclusion

It should be highlighted that the Delivery and Birth Plan is positively related with increased "skin to skin contact", "late clamping of the umbilical cord" and the rate of "eutocic births", practices that directly and indirectly reduce the health spending on hospitalization rates of women and infants. In addition, the women's autonomy is strengthened by the "choice of the dilation and delivery position", the "intake of foods or fluids" and even through the use of "enemas" and the "perineal shaving".

In that sense, the Delivery and Birth Plan positively influences the delivery process and its outcome, enhancing the dimensions of the women's safety, effectiveness and satisfaction, as well as their empowerment.

Further research is needed to determine the cause of the low number of Delivery and Birth Plans in the study context. In addition, health policies need to be created for the dissemination of these documents and the use of the Delivery and Birth Plans needs to be enhanced among the pregnant women in the community studied. In that sense, the Primary Health Care midwife is the competent professional to accompany the women during the elaboration of this document.
